# Clinical features and viral quasispecies characteristics associated with infection by the hepatitis B virus G145R immune escape mutant

**DOI:** 10.1038/emi.2017.2

**Published:** 2017-03-22

**Authors:** Yuan Xue, Ming-Jie Wang, Zhi-Tao Yang, De-Min Yu, Yue Han, Dao Huang, Dong-Hua Zhang, Xin-Xin Zhang

**Affiliations:** 1 Clinical Virology Research Laboratory, Ruijin Hospital, Shanghai Jiaotong University School of Medicine, Shanghai 200025, China; 2 Department of Infectious Diseases, Institute of Infectious and Respiratory Diseases, Ruijin Hospital, Shanghai Jiaotong University School of Medicine, Shanghai 200025, China; 3 Pôle Sino-Français de Recherches en Science du Vivant et Génomique, Ruijin Hospital, Shanghai Jiaotong University School of Medicine, Shanghai 200025, China; 4 Translational Medicine Research Center, Ruijin Hospital North, Shanghai Jiaotong University School of Medicine, Shanghai 201800, China

**Keywords:** deep sequencing, genetic heterogeneity, hepatitis B virus, mutation

## Abstract

Coexistence of the hepatitis B surface antigen (HBsAg) and hepatitis B surface antibody (anti-HBs) is an uncommon phenomenon, and the underlying mechanisms remain largely unknown. Amino-acid (aa) substitution from glycine to arginine at aa 145 (G145R), in the major hydrophilic region, has been reported in patients with HBsAg and anti-HBs coexistence. However, there is limited knowledge about the clinical features and viral quasispecies characteristics associated with G145R mutant hepatitis B virus (HBV) infection. We herein describe the dynamic changes in the serological and virological markers in a case of hepatitis B with coexisting HBsAg and anti-HBs, caused by a G145R immune escape mutant (genotype C). Entecavir was administered during the 4th week after admission. Alanine aminotransferase peaked in the 16th week, while both the HBsAg and HBeAg declined rapidly. HBsAg clearance and hepatitis B e antigen (HBeAg)/hepatitis B e antibody (anti-HBe) seroconversion were achieved in the 36th week, and then entecavir was withdrawn. A follow-up of 96 weeks showed that HBV DNA remained undetectable and that anti-HBs was maintained above 100 mIU/mL. The quasispecies characteristics of the G145R mutant HBV were investigated via ultra-deep sequencing. The complexity and genetic distance of the S and RT regions were much higher in the 8th week than at baseline or in the 4th week. Moreover, the frequencies of mutations (L173P, Q181R and A184V) in cytotoxic T lymphocyte epitopes increased before entecavir treatment. These findings extend understanding of the evolution of HBV under host immune pressure and of the clinical outcomes of affected patients.

## INTRODUCTION

The hepatitis B surface antigen (HBsAg) is a primary element used for the diagnosis of hepatitis B virus (HBV) infection, whereas hepatitis B surface antibody (anti-HBs) are often used as a serological marker of recovery. Nevertheless, the coexistence of HBsAg and anti-HBs has been reported,^1, 2, 3, 4^ although the mechanisms underlying this uncommon phenomenon remain unclear. Mutations in the ‘α' determinant of the major hydrophilic region (MHR), such as amino-acid (aa) substitution from glycine to arginine at aa 145 (G145R), have been reported in patients with coexistence of HBsAg and anti-HBs.^2, 5, 6, 7^ However, there is limited knowledge about the clinical features and quasispecies characteristics associated with infection by G145R mutant HBV.

We herein describe the dynamic changes in the serological and virological markers in a case of hepatitis B with coexisting HBsAg and anti-HBs caused by a G145R immune escape mutant. Moreover, the quasispecies characteristics, including the frequency of mutations in full-length HBV genomes, were investigated using ultra-deep sequencing.

## MATERIALS AND METHODS

### Patient

The patient was a 90-year-old Chinese man who was admitted to the Geriatric Ward of Ruijin Hospital on 2 September 2014. He had not been vaccinated against HBV. A health checkup performed in October 2009 showed that he was negative for the HBsAg, hepatitis B e antigen (HBeAg) and hepatitis B e antibody (anti-HBe), whereas he was positive for the hepatitis B core antibody (anti-HBc, 1.41S/CO) and anti-HBs (129.57 mIU/mL, with a protective value above 10 mIU/mL). He had no history of known risk factors for HBV infection. He had been diagnosed with a gastric ulcer and diabetes three years before admission. The clinical and virological data from the patient were obtained during 2 years of follow-up. Informed consent was obtained from the patient.

### Serological, biochemical and virological tests

Serological markers of HBV were quantified using an enzyme immunoassay kit (Murex Abbott, Chicago, IL, USA). HBV DNA was detected with a Cobas TaqMan HBV Test version 2.0 (lower limit of detection, 20 IU/mL; Roche Diagnostic, Basel, Switzerland). The alanine aminotransferase (ALT) level was determined according to the manufacturer's protocol.

### DNA extraction and direct PCR sequencing

Serum was obtained from the patient during the first (week 0; baseline), 4th and 8th weeks after admission. HBV DNA was extracted using a QIAamp DNA Blood Mini Kit (Qiagen, Tokyo, Japan). The S gene was amplified by nested PCR and sequenced after purification. The nucleotide sequences were aligned with reference sequences retrieved from GenBank, and the HBV genotype was identified through online software analysis (http://www.ncbi.nlm.nih.gov/projects/genotyping/formpage.cgi).

### Ultra-deep sequencing

The entire HBV genomes were amplified with nine overlapping fragments (p1–p9), and then the concentration of the PCR products was measured using a Qubit dsDNA HS Assay Kit (Invitrogen, Carlsbad, CA, USA). A library of PCR products was established using a Nextera DNA Sample Prep Kit (Illumina, San Diego, CA, USA). Sequencing of the PCR products was performed using an Illumina (San Diego, CA, USA) Miseq system, according to the manufacturer's paired-end 2 × 300 bp protocol. Image analysis and base calling were performed using Illumina CASAVA software version 1.8.2 with default parameters.

### Treatment of sequencing data

Raw reads were pre-processed using CutAdapt^8^ version 1.9.1 to cut adaptor sequences and trim low-quality reads (length <250 bp or base quality <30). Filtered read pairs were aligned to the HBV reference genome sequence (GenBank Accession Number: AB014381) with Bowtie2 version 2.2.6.^9^ Samtools version 0.1.19 was used to generate coordinate-sorted bam files.^10^ Sequencing errors induced by PCR and ultra-deep sequencing would complicate the analysis of mixed populations and result in inflated estimates of genetic diversity; therefore, we used ShoRAH 0.5.1 software,^11^ which applied a probabilistic Bayesian approach to minimize the effects of errors.

### Sequence characteristics and statistical analysis

The virus quasispecies heterogeneity was mainly evaluated on the basis of complexity and diversity. The quasispecies complexity was measured using normalized Shannon entropy (Sn). The Sn can be calculated with a previously described formula.^12, 13^ The Sn of regions p1–p9 were calculated using Perl scripts in Perl 5.20. The quasispecies diversity was evaluated on the basis of the mean genetic distance (*d*). MEGA^14^ version 6.0 was used to calculate the genetic distance of all regions by using the Tamura three-parameter model.

Variations were detected and analyzed using Perl scripts. High-confidence variations with a frequency ≥1% of the total viral population were selected and analyzed. The quasispecies characteristics of the HBV genome were analyzed at baseline and during the 4th and 8th weeks as described previously.^15^

## RESULTS

### Dynamic changes in clinical characteristics during infection by G145R mutant HBV

**Figure 1:**
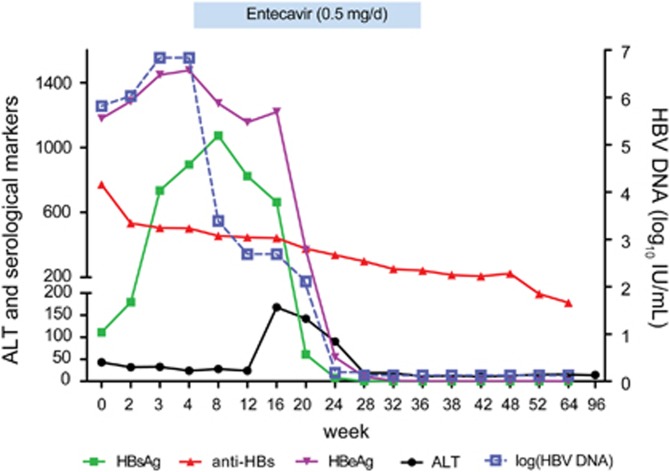
The dynamic changes in serological markers, HBV DNA and ALT during infection by the HBV G145R immune escape mutant**.** Entecavir treatment was started during the 4th week. HBsAg became undetectable, and HBeAg/anti-HBe seroconversion was achieved during the 36th week, and then entecavir was withdrawn. aminotransferase, ALT; hepatitis B surface antigen, HBsAg; hepatitis B virus, HBV.

At the time of admission, laboratory tests were positive for HBsAg (111.32 IU/mL), anti-HBs (771.71 mIU/mL), HBeAg (1179.194S/CO), anti-HBc IgM (1.21S/CO), and HBV DNA (5.82 log_10_ IU/mL) and indicated a slight elevation in ALT (43 IU/L; Figure 1). On 30 September 2014 (the 4th week after admission), tests revealed that the HBsAg, HBeAg and HBV DNA levels had increased significantly (895 IU/mL, 1475.496 S/CO and 6.84 log_10_ IU/mL, respectively), whereas the anti-HBs level had decreased to 502 mIU/mL. On the basis of the elevated HBV DNA and HBeAg levels, entecavir treatment was initiated at 0.5 mg per day with the patient's consent.

Interestingly, the ALT level increased to 168 IU/L during the 16th week, whereas the levels of HBsAg and HBeAg declined rapidly. HBV DNA became undetectable on 25 February 2015 (in the 24th week). At that time, the HBsAg and HBeAg levels had also decreased significantly (7.86 IU/mL and 54.728S/CO, respectively). HBsAg became undetectable, and HBeAg/anti-HBe seroconversion was achieved during the 36th week, and then entecavir was withdrawn. A follow-up of 96 weeks showed that HBV DNA remained undetectable and that the anti-HBs level was maintained above 100 mIU/mL. As shown in Figure 1, the ALT level remained normal until the end of the 96-week follow-up.

### Evolution of the viral quasispecies during G145R mutant HBV infection

**Figure 2:**
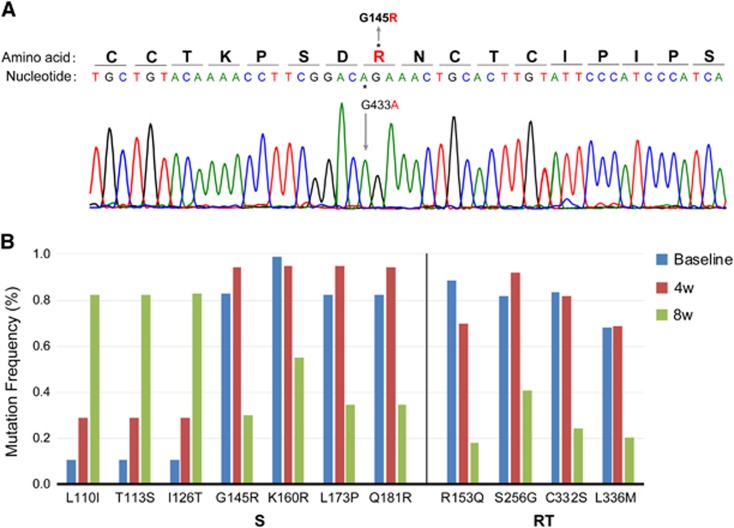
The G145R mutation (**A**) and dynamic changes in major mutations in the S and RT regions (**B**). (**A**) Amino-acid substitution from glycine to arginine at aa 145 (G145R) in the major hydrophilic region of HBsAg was detected by direct sequencing at the time of admission. (**B**) The frequencies of major mutations at baseline and during the 4th and 8th weeks were detected using ultra-deep sequencing. Hepatitis B surface antigen, HBsAg.

**Figure 3:**
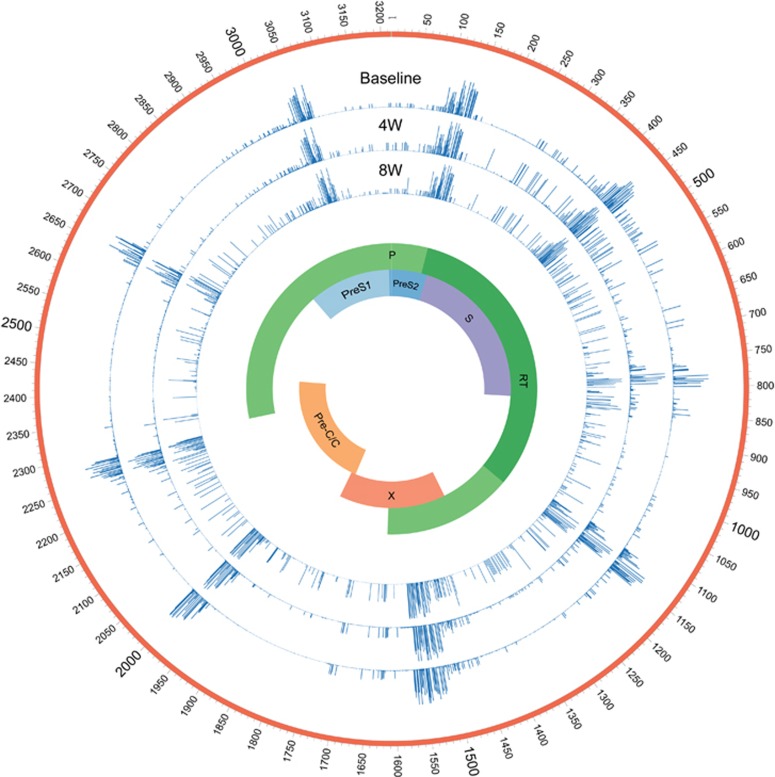
The complexity of the full-length HBV genome at baseline and during the 4th and 8th weeks. The complexity for each nucleotide was evaluated using ultra-deep sequencing. Entecavir treatment was initiated during the 4th week. Blue bars indicate the complexity for each nucleotide in the full-length HBV genome. Hepatitis B virus, HBV.

A direct DNA sequence analysis was performed, and HBV genotype C with a G145R mutation was confirmed at the time of admission (Figure 2A). Accordingly, the patient was diagnosed with hepatitis B caused by a G145R immune escape mutant. Ultra-deep sequencing showed that the complexity and genetic distances within the S and RT regions in the 8th week were much higher than those at baseline and in the 4th week (Figure 3, Table 1).

### Dynamic changes in the mutations in the HBV genome

The mutations with frequencies that changed during the course of treatment are shown in Figure 2B and the Supplementary Table S1. In addition to G145R, new dominant and minor mutations were detected. The frequencies of four mutations in the S region (G145R, K160R, L173P and Q181R), four mutations in the RT region (R153Q, S256G, C332S and L336M) and five mutations in the X region (S54P, L55F, R56P, L58P and C69P) decreased in the 8th week, whereas the frequencies of many mutations in the S, P and X regions clearly increased. The mutations with an increased frequency of detection were much more common than those with a decreased frequency (Supplementary Figure S1).

With regard to the ‘hot-spot' mutations in the HBV genome, G1896A in the preC region and A1762T/G1764A in the basic core promoter region were studied. We also screened several mutations relevant to antiviral resistance, such as I169L, L180M, A181V, T184I, S202C, M204V/I, N236T and M250I in the RT region. None of these mutations were detected before or after the administration of entecavir.

### Dynamic changes in the immune epitopes in the S region

As shown in Supplementary Figure S1, the frequencies of mutations (L173P, Q181R and A184V) in cytotoxic T lymphocyte (CTL) epitopes^16, 17^ were increased before entecavir treatment, whereas mutations in the CTL domain aa 44–59 unexpectedly increased after entecavir treatment. Unlike mutations in the CTL domain, Y221C in the T-helper cell domain^16^ exhibited a high frequency both before and after entecavir treatment.

## DISCUSSION

In the present study, the clinical and quasispecies characteristics associated with G145R immune escape mutant HBV infection were described in detail. The full-length HBV sequences obtained by ultra-deep sequencing showed that the complexity and genetic distances of the S and RT regions were much higher in the 8th week than at baseline and in the 4th week. Mutations in the MHR of HBsAg and T-cell epitopes may contribute to immune escape.

On the basis of the level of protective anti-HBs (129.57 mIU/mL) observed in 2009 and recent serological changes, hepatitis B caused by the G145R immune escape mutant was diagnosed. Infection with G145R mutant HBV may involve a clinical onset that is insidious, as in our subject, who had a slightly elevated ALT level. Of note, HBsAg and anti-HBs coexisted for 36 weeks in this patient. The prevalence of this atypical serological pattern in HBsAg-positive carriers is 2.9%–5%,^5, 18^ and it tends to occur in older carriers (aged over 40 years) more often than in carriers who are HBsAg-positive but anti-HBs negative.^19^ Another study has shown that patients with coexisting HBsAg and anti-HBs had higher proportions of HBV genotype C than patients with HBsAg-positive but anti-HBs-negative infections.^5^ In addition, carriers with coexisting HBsAg and anti-HBs have high HBeAg- and HBV DNA-positive rates and are therefore infectious. A retrospective cohort study has shown that HBeAg and HBV DNA persist for several decades in most carriers, and their risk of hepatocellular carcinoma (HCC) is increased.^20^ Fortunately, the HBsAg disappeared and HBeAg seroconversion was achieved in this patient, who eventually cleared the HBV and recovered.

The mechanisms underlying the simultaneous appearance of HBsAg and anti-HBs remain controversial. The presence of amino-acid substitutions in the MHR of HBsAg, such as G145R and I126T, can change the immunogenicity of HBsAg, thus resulting in HBsAg/anti-HBs coexistence and immune escape. Consequently, these mutations can also lead to the diagnostic failure of commercial assays for HBsAg, as well as to the prophylactic failure of immunoglobulin or vaccines. The G145R mutation in the S region is likely to be responsible for weak recognition by anti-HBs.^21, 22^ Because the G145R mutation alters the projecting loop of the ‘α' determinant, pre-existing neutralizing antibodies cannot adequately recognize the changed epitope. Other studies have suggested that exposure to different subtypes of HBV accounts for the anomalous serological changes observed in these patients.^3, 19, 23^ Zhang *et al.*^3^ have reported that the subtype-specific anti-HBs in these patients are directed to HBsAg subtypes other than the coexisting subtype and are unable to neutralize the coexisting HBsAg. Owing to a high replication rate and lack of proofreading activity during reverse transcription, HBV exists as a spectrum of variants called quasispecies.^12, 13^ These variants are genetically distinct but closely related. Because of their different adaptability, the presence and type of quasispecies are related to the outcome of HBV infection. In the present study, data from ultra-deep sequencing revealed that mutants harboring different mutations, including G145R and I126T, coexisted in the patient. Furthermore, the ratio of coexisting mutants varied during the infection. These findings are consistent with the two aforementioned hypotheses and suggest that mutations in the S gene, as well as different subtypes of HBV, may play important roles in the coexistence of HBsAg and anti-HBs.

Increasing evidence indicates that the characteristics of HBV quasispecies, including their complexity and genetic distances, are related to the outcome of HBV infection and antiviral therapy.^12, 13, 24, 25, 26^ However, the characteristics of the entire genomes of HBV quasispecies determined using ultra-deep sequencing during G145R mutant infection have not yet been reported. The quasispecies heterogeneity within the S and RT regions had not changed significantly during the 4th week compared with baseline but clearly increased 1 month after the onset of administration of entecavir.

Importantly, ultra-deep sequencing provided insights into the selection process of immune escape mutants during HBV infection and entecavir treatment. Accompanying high levels of HBV DNA, the frequencies of G145R and other mutations (K160R, L173P, Q181R in the S region and R153Q, S256G, C332S, L336M in the RT region) were high both at baseline and in the 4th week. Interestingly, under antiviral therapy, both the HBV DNA level and the frequencies of the aforementioned mutations declined during the 8th week, as expected. In contrast, the frequencies of L110I, T113S and I126T, which may be compensatory mutations, increased in the S region. Compared with other antiviral agents, entecavir is more potent, and it causes higher selective pressure on HBV quasispecies, thus possibly accounting for the elevated quasispecies complexity observed in this study. High quasispecies complexity, which is associated with a large reservoir of variants and more complementary interactions among variants, means that there is high quasispecies adaptability.^12^ In addition, the interplay between different mutants may facilitate the replication of the viral population.^27^ Thus, quasispecies with higher complexity should theoretically tend to breach genetic barriers, thus leading to drug resistance. Nevertheless, entecavir has a higher genetic barrier to drug resistance, and the use of entecavir for therapy can prevent the emergence of many of the known mutations leading to drug resistance. To date, the consequences of this selection and evolution remain largely unknown.

A previous study has indicated that mutations in the S region mainly occur in the N-terminal region (aa 1–99) and the MHR (aa 100–169), which contain the ‘α' determinant (aa 124–147), rather than the C-terminal region (aa 170–226).^5^ Consistently with results from a previous study,^5^ the data from ultra-deep sequencing in the present study showed that mutations in the S region were mainly located in the MHR and N-terminal region, both before and after antiviral therapy. The most frequently observed mutations in the S region were located at aa 126, 129, 130, 133, 145 and 181.^2, 6, 22^ The G145R mutation in the MHR, which is one of the most prevalent immune escape mutations, can impair HBsAg secretion^28^ and has been reported in patients with advanced liver diseases and even liver cancer.^15^ In addition, the Q181R mutation has been recently reported in a new vaccine escape mutant.^22^ Some of these mutations have been reported in cases of occult HBV infection.^2, 5, 6^

In summary, our study shows the dynamic changes in the clinical features and quasispecies characteristics during infection with G145R mutant HBV. In addition to mutations in the MHR of HBsAg, mutations in T-cell epitopes may contribute to immune escape. These findings obtained by ultra-deep sequencing extend understanding of the evolutionary pattern of HBV under host immune pressure and of the clinical outcomes of patients.

## Figures and Tables

**Table 1:** The genetic distances at the nucleotide level at basline and during the 4th week and 8th week (10^−3^ substitutions/site)

Fragments	Region	0w	4^th^w	8^th^w
p1	200–605	23.65054	39.21177	41.7487
p2	542–994	17.895	18.9791	58.4646
p3	894–1368	9.82769	12.26898	36.0831
p4	1306–1803	21.67005	19.87999	36.3433
p5	1746–2108	4.90833	5.42481	23.0531
p6	2026–2471	5.999	10.40294	34.128
p7	2413–2815	5.59832	8.48556	24.6503
p8	2813–3135	7.97107	11.18413	20.7532
p9	1–252, 3097–3125	17.27809	20.95363	19.9135
