# Supplementary material for: Clinical features and viral quasispecies characteristics associated with infection by the hepatitis B virus G145R immune escape mutant
Source: Emerg Microbes Infect. 2017 Mar 22;6(3):e15–. doi: 10.1038/emi.2017.2 (PMC5378923; doi:10.1038/emi.2017.2)
Supplement: Supplementary Figure S1 [file emi20172x1.pdf]

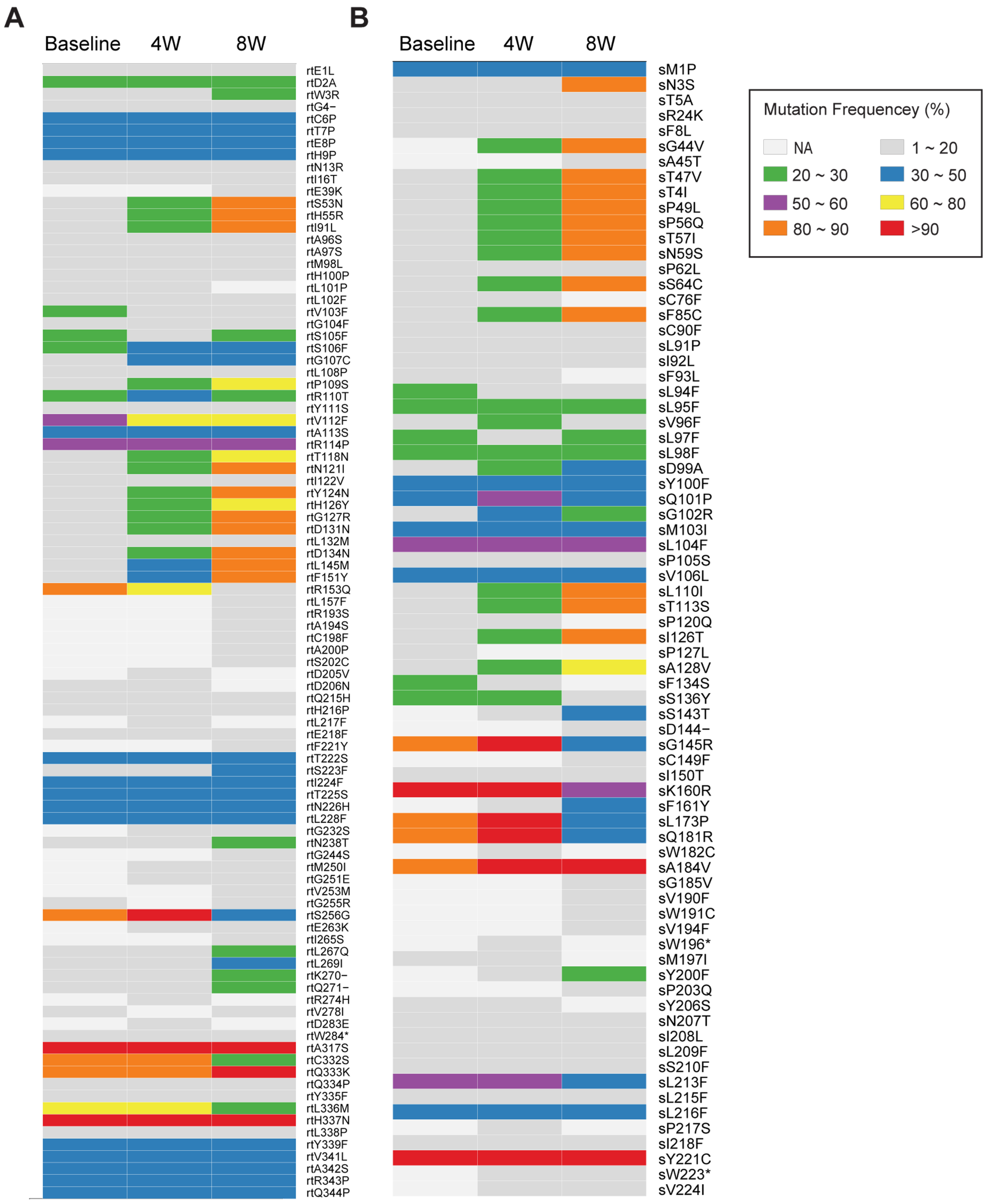

Supplementary Figure S1

The frequencies of nucleotide (A) and amino acid (B) variations in the HBV S region.
